# *Synechococcus* Assemblages across the Salinity Gradient in a Salt Wedge Estuary

**DOI:** 10.3389/fmicb.2017.01254

**Published:** 2017-07-06

**Authors:** Xiaomin Xia, Wang Guo, Shangjin Tan, Hongbin Liu

**Affiliations:** Division of Life Science, The Hong Kong University of Science and TechnologyHong Kong, Hong Kong

**Keywords:** salt wedge estuary, pyrosequencing, *rpoC1* gene, *cpcBA* operon, salinity gradient

## Abstract

*Synechococcus* are the most abundant and widely distributed picocyanobacteria in the ocean. The salt-wedge type of estuary possesses the complete horizontal and vertical gradient of salinity together with other physical and chemical parameters. In order to reveal whether such a complex environmental gradient harbors a high diversity of *Synechococcus*, we investigated the abundance, taxonomic composition and pigment genetic diversity of *Synechococcus* in surface and bottom waters across the salinity gradient in a salt-wedge estuary by flow cytometric analysis and pyrosequencing of the *rpoC1* gene and *cpcBA* operon (encoding phycocyanin). *Synechococcus* were ubiquitously distributed in the studied region, with clear spatial variations both horizontally and vertically. The abundance and diversity of *Synechococcus* were low in the freshwater-dominated low salinity waters. By pyrosequencing of the *rpoC1* gene, we have shown that with the increase of salinity, the dominant *Synechococcus* shifted from the freshwater *Synechococcus* to the combination of phylogenetic subcluster 5.2 and freshwater *Synechococcus*, and then the strictly marine subcluster 5.1 clade III. Besides, the composition of *Synechococcus* assemblage in the deep layer was markedly different from the surface in the stratified waters (dissimilarities: 40.32%-95.97%, SIMPER analysis). High abundance of clade III *Synechococcus* found in the brackish waters may revise our previous understanding that strains of this clade prefers oligotrophic environment. Our data also suggested that both the phylogenetic subcluster 5.3 *Synechococcus*, a lineage that was not well understood, and subcluster 5.1 clade I, a typical cold water lineage, were widely distributed in the bottom layer of the estuary. Clade I detected in the studied region was mainly contributed by subclade IG. Analysis of the *cpcBA* operon sequences revealed niche partitioning between type 1 and type 3 *Synechococcus*, with type 2 distributed broadly across the whole environmental gradients. Our results suggest that the salt wedge estuary provides various niches for different lineages of *Synechococcus*, making it an environment with high *Synechococcus* diversity compared with adjacent freshwater and shelf sea environments.

## Introduction

*Synechococcus* is one of major components of the phytoplankton community in both marine (Glover et al., 1986; Partensky et al., 1999) and freshwater (Callieri and Stockner, 2002; Sarmento et al., 2008) ecosystems. Compared with *Prochlorococcus*, another important pico-cyanobacteria which dominate in the oligotrophic open ocean, *Synechococcus* have higher nutrient requirements and are therefore more abundant in coastal (Li, 1998; Flombaum et al., 2013) and upwelling waters (Partensky et al., 1999; Cuevas and Morales, 2006). For instance, the highest *Synechococcus* abundance was recorded in the Costa Rica Dome where strong upwelling occurs (Saito et al., 2005), varying between 1.2 × 10^6^ and 3.7 × 10^6^ cells mL^-1^. A high abundance of *Synechococcus* was also reported in the Red Sea (Veldhuis and Kraay, 1993), Baltic Sea (Kuosa, 1991), and Chesapeake Bay (Wang et al., 2011). In Hong Kong coastal waters, *Synechococcus* are also important primary producers in the summer, with the maximum abundance reaching 5.7 × 10^5^ cells mL^-1^ (Liu et al., 2014).

*Synechococcus* are divided into three major pigment types by their different phycobiliprotein compositions: type 1 binds only phycocyanobilin (PCB), type 2 binds both PCB and phycoerythrobilin (PEB), while type 3 contains PCB, PEB and phycourobilin (PUB) (Six et al., 2007). Hence, type 1 is also called PC-only *Synechococcus* while type 2 and 3 are PE-containing *Synechococcus*. Type 3 is further divided into four subtypes, 3a (low PUB), 3b (medium PUB), 3c (high PUB) and 3d (variable PUB), according to the compositional proportion of PUB relative to PEB. Studies have widely reported different geographical distributions of *Synechococcus* pigment types – type 1 is abundant in high nutrient and turbidity coastal and estuarine waters, type 2 prefers relatively clean coastal waters, while type 3 dominates in oligotrophic open ocean (Olson et al., 1988, 1990; Wood et al., 1998). Besides *in situ* fluorometer (Cowles et al., 1993) and FCM (Olson et al., 1988), recently molecular methods have been applied to study *Synechococcus* pigment diversity in marine waters (Crosbie et al., 2003; Haverkamp et al., 2009; Everroad and Wood, 2012; Liu et al., 2014; Xia et al., 2017). The *cpeBA* operon and *cpcBA* operon are two gene markers commonly used to identify *Synechococcus* pigment types (Crosbie et al., 2003; Haverkamp et al., 2008; Liu et al., 2014; Xia et al., 2017).

Taxonomically, cluster 5 marine *Synechococcus* is further divided into three subclusters, S5.1, S5.2 and S5.3 according to the gene markers, such as the *16S rRNA* and *rpoC1* (Herdman et al., 2001; Fuller et al., 2003; Mazard et al., 2012). These three subclusters are further composed of at least 19 phylogenetical lineages (Farrant et al., 2016; Xia et al., 2017). Studies that adopted culture independent methods have revealed niche differentiation in *Synechococcus* lineages (Zwirglmaier et al., 2008; Huang et al., 2012; Xia et al., 2017). For example, clade I is known as cold water *Synechococcus*, while clade II is dominant in tropical/subtropical warm waters. Previous studies have also reported that distinct *Synechococcus* communities were present in the oligotrophic oceanic waters and nutrient rich coastal waters (Scanlan and West, 2002). Environmental factors such as concentration and type of inorganic nitrogen (Ahlgren and Rocap, 2006), phosphate concentration (Tetu et al., 2009), temperature (Pittera et al., 2014), salinity (Rajaneesh and Mitbavkar, 2013; Xia et al., 2015), and trace metal (Ahlgren et al., 2014) are all known to influence the distribution of *Synechococcus* lineages. However, the niches of some *Synechococcus* lineages remain unknown.

Previous studies suggested that *Synechococcus* pigment genes, such as PE-encoding genes, have undergone horizontal gene transfers between *Synechococcus* lineages during the evolution of this genus (Six et al., 2007; Everroad and Wood, 2012). This makes it impossible to identify a *Synechococcus* taxonomic lineage and pigment type at the same time based on a single gene marker. For examples, the phylogenetic tree based on the *cpeBA* operon sequences clearly grouped several *Synechococcus* lineages (see Figure 3 in Everroad and Wood, 2012). On the other hand, some lineages are composed by different pigment types. For example, clade II *Synechococcus* have at least 4 pigment types: type 2, 3a, 3c, and 3d ^1^ (Roscoff *Syenchococcus* database). Hence, different from identification of *Synechococcus* pigment types which is based on *cpeBA* and *cpcBA* operon, taxonomic lineage of a *Synechococcus* is classified via housekeeping genes, such asITS (Haverkamp et al., 2008), 16s rRNA gene (Fuller et al., 2003), *rpoC1* (Mühling et al., 2006), and *petB* (Farrant et al., 2016).

*Synechococcus* community composition in estuaries or river plumes is often distinct from that in saline waters. A study conducted in Hong Kong water has shown that the water influenced by freshwater discharge from the Pearl River is dominated by PC-only (type 1) S5.2 *Synechococcus*, freshwater *Synechococcus*, and *Cyanobium*, while the coastal water not directly impacted by the river plume is dominated by various clades of marine *Synechococcus* S5.1 (Xia et al., 2015). The study also suggested that *Synechococcus* imported by the freshwater discharge are an important component of the cyanobacterial phytoplankton in the estuarine ecosystems. Similar observation was also reported by the studies carried out in the Zuari estuary and Changjiang estuary (Rajaneesh and Mitbavkar, 2013; Chung et al., 2015).

Due to high nutrient inputs, estuaries often sustain high levels of productivity. Salt wedge estuaries with strong vertical salinity gradient harbor different microbial communities in the surface and deep water (Korlević et al., 2016). The Pearl River is one of the largest rivers in China with a typical salt wedge estuary in the wet season (Harrison et al., 2008). In contrast to the increasing salinity along the river-estuary-coastal water transition, nutrient concentrations gradually decrease (Harrison et al., 2008). The strong gradient of environmental conditions makes the Pearl River estuary an ideal place to evaluate factors affecting the spatial distribution of *Synechococcus* lineages. However, till now, no study of *Synechococcus* phylogenetic diversity and pigment diversity along the salinity gradient with different depths was conducted in this strongly stratified estuary.

In order to study *Synechococcus* abundance, community taxonomic composition and pigment diversity in the salt wedge estuary, we conducted a cruise in July 2014 to collect samples along a salinity gradient in the Pearl River-estuary-coast system. Abundance of *Synechococcus* was evaluated by flow cytometric analysis. *Synechococcus* taxonomic composition and pigment diversity were assessed through pyrosequencing of the *rpoC1* gene and *cpcBA* operon, respectively. The relationship between environmental factors and *Synechococcus* diversity was also analyzed.

## Materials and Methods

### Sample Collection

**FIGURE 1:**
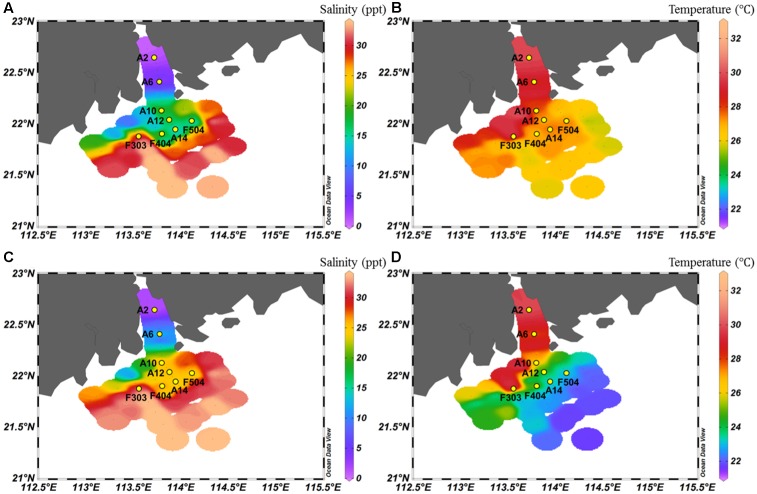
Salinity **(A,C)** and temperature **(B,D)** of surface **(A,B)** and bottom **(C,D)** water of the study area.

Samples were collected from the Pearl River estuary on a cruise conducted from 13 to 20 July 2014 (**Figure 1** and **Table 1**). Salinity, temperature and depth were measured by a conductivity-temperature-depth rosette system (CTD, Sea Bird Electronics). At each station, 0.5–1 L of water was collected from surface and bottom (1 m above the bottom) layers, pre-filtered through a 3.0 μm (47 mm) polycarbonate membrane (PALL Corporation) and then filtered onto a 0.22 μm (47 mm) polycarbonate membrane for DNA extraction. Membranes were frozen at –80°C immediately after filtration. For counting *Synechococcus* abundance, 1.8 mL water from each station was fixed with seawater buffered paraformaldehyde (0.5%, final concentration), flash frozen in liquid nitrogen and stored at –80°C. Water samples for nutrient measurement were filtered with 0.45μm cellulose acetate membranes and were stored at -20°C until analysis. Analytical protocols for nutrients followed Dai et al. (Dai et al., 2008). The method detection limits are 0.5 μM for ammonia, 0.02 μM for nitrite, 0.07 μM for nitrate, and 0.17 μM for phosphate.

**Table 1:** Characteristics of sampling sites and measured environmental factors.

Sample	Station	Latitude [N]	Longitude [E]	Sampling depth (m)	Temperature (°C)	Salinity (ppt)	NH_4_^+^ (μmol L^-1^)	NO_2_^-^ (μmol L^-1^)	NO_3_^-^ (μmol L^-1^)	PO_4_^3-^ (μmol L^-1^)
A2S	A2	22.6528	113.7168	0.5	30.06	1.4	2.14	2.34	134.52	1.44
A2B	A2	22.6528	113.7168	12	30.06	1.6	3.58	2.41	135.52	1.48
A6S	A6	22.4118	113.7701	0.5	29.07	3.3	5.6	5.29	107.84	1.26
A6B	A6	22.4118	113.7701	28	27.79	23.5	4.57	5.47	40.91	0.96
A10S	A10	22.1377	113.7943	0.5	29.02	19.5	6.84	3.67	55.65	0.73
A10B	A10	22.1377	113.7943	18	26.98	32.2	5.82	2.69	8.83	0.68
A12S	A12	22.0406	113.8739	0.5	29.87	17.2	0.84	3.7	61.31	0.66
A12B	A12	22.0406	113.8739	9	24.23	34	0.5*	1.17	7.21	0.51
A14S	A14	21.9639	113.9376	0.5	32.02	14.4	4.55	0.88	50.62	0.17*
A14B	A14	21.9639	113.9376	7	23.27	34	0.79	1.97	4.01	0.32
F504S	F504	22.0388	114.1124	0.5	31.72	15.50	0.5*	0.71	45.78	0.17*
F504B	F504	22.0388	114.1124	27.0	22.98	34.70	0.5*	1.30	2.41	0.25
F404S	F404	21.9040	113.7789	0.5	29.11	19.70	0.5*	3.27	52.83	0.17*
F404B	F404	21.9040	113.7789	27.0	24.87	34.20	0.5*	0.77	7.59	0.53
F303S	F303	21.8837	113.5500	0.5	29.52	30.10	0.5*	0.68	5.42	0.17*
F303B	F303	21.8837	113.5500	21.0	29.32	33.20	0.5*	0.14	0.67	0.17*
F603S	F603	22.0326	114.3365	0.5	29.08	33.20	0.5*	0.13	1.74	0.17*
F603B	F603	22.0326	114.3365	35.0	22.08	34.40	0.5*	0.31	3.21	0.35

*^∗^Lower than the limit of detection*.

### Analysis of *Synechococcus* Abundance

*Synechococcus* cells were enumerated using a Becton-Dickson FACSCalibur flow cytometer equipped with dual lasers of 488 and 635 nm with a high flow rate (Liu et al., 2014). Ten microliter yellow–green fluorescent beads (1 μm, Polysciences, Warrington, PA, United States) were added to each sample as an internal standard. Flow cytometric data were analyzed using WinMDI software 2.9 (Joseph Trotter, Scripps Research Institute, LaJolla, CA, United States). PC-only and PE-containing type *Synechococcus* were counted following the method described by Liu et al. (2014). However, samples from F303 were lost.

### DNA Extraction, PCR, and Sequencing

Genomic DNA was extracted using the PureLink Genomic DNA mini kit (Invitrogen, CA, United States) and was eluted in TE buffer (Tris-EDTA buffer: 10 mM Tris,1 mM EDTA,pH8.0). For amplification of the *rpoC1* gene, the PCR followed the protocol of Mühling et al. (2006). The first round of PCR used the primer *rpoC1*-N5 and the C-terminal primer *rpoC1*-C, and the PCR products were used as templates for a second round of PCR with modified primer *rpoC1*-39F (5′-adaptor+barcode+GGNATNGTNTGYGAGCGYTG) and *rpoC1*-462R (5′-adaptor+CGYAGRCGCTTGRTCAGCTT) (Xia et al., 2015). The PCR products were gel-purified using the Qiaquick gel purification kit (Qiagen, Hilgen, Germany) as described by the manufacturer. Purified amplicons were sequenced using the GS Junior pyrosequencing system according to manufacturer instructions (Roche, 454 Life Sciences, Branford, CT, United States).

For amplification of the *cpcBA* operon sequences, we used the primer pair SyncpcB-Fw (5′-adaptor+barcode+ATGGCTGCTTGCCTGCG-3′) and SyncpcA-Rev (5′-adaptor +ATCTGGGTGGTGTAGGG-3′) designed by Haverkamp et al. (2008). The PCR reaction mixture was composed of 1 μL of template DNA, 2.5 μL of 10× PCR buffer, 0.5 μL of 10 mM dNTP mixture, 0.75 μL of 50 mM MgCl_2_, 1 unit of Platinum taq DNA polymerase (Invitrogen, CA, United States), and 1 μL of each forward and reverse primer (10 nM). Sterile MilliQ-grade water was added to a final reaction volume of 25 μL. The PCR reactions were run on a Bio-Rad PCR machine. The program was 5 min at 94°C, followed by 40 cycles of 30 s at 94°C, 30 s at 55°C and 1 min at 72°C. The final elongation step was 10 min at 72°C. The PCR products were gel purified and sequenced in the GS Junior 454 sequencing system.

### 454 Post-run Sequence Analyses

Analysis of the *rpoC1* and *cpcBA* sequence was conducted using the microbial ecology community software program Mothur^2^ (Schloss et al., 2009). Raw sequences were first processed by removing barcodes and primers, then only reads with an average quality score above 25 and length longer than 300 nt were taken into account. Sequences were then denoised using the command *shhh.seqs* with sigma value of 0.01. Sequences containing ambiguous bases and homopolymer longer than 8 bp were also screened. Chimeras were identified using the command *chimera.uchime* and were then removed. After the above quality control, sequences were identified by local Blast using BioEdit with an expectation value 0.01 (Hall, 1999). For the analysis of *rpoC1* gene, sequences classified as *Prochlorococcus* and *Synechocystis* were removed, and the remaining sequences that were less than 90% identical to the S5.1 clades and 85% identical to S5.2, S5.3, *Cyanobium*, and FS reference sequences (Supplementary Table S1) were assigned as unclassified (Xia et al., 2015). Similarly, for the *cpcBA* operon, sequences were identified by the local blast with the expectation value 0.01. The reference sequences of the *rpoC1* (Xia et al., 2015) and *cpcBA* operon were listed in Supplementary Tables S1, S2. The *cpcBA* operon reference sequences were from the NCBI GenBank database^3^ and the pigment type of representative strains was determined according to the Roscoff *Synechococcus* database ^4^ and Everroad and Wood’s work (Everroad and Wood, 2012). As there were three copies of the *cpcBA* operon in the genomic sequence of type 1 *Synechococcus* (Six et al., 2007), the number of resulting type 1 sequences was divided by three in calculating the relative abundance of each *Synechococcus* pigment type. Coverge and operational taxonomic units (OTUs) numbers were calculated at the cutoff level of 3% for the *rpoC1* gene and 5% for the *cpcBA* operon using Mothur’s command *summary.single*. OTUs which contain only 1 sequence were removed. The relative abundance of each OTU in a sample was calculated using the command *get.relabund*. The Margalef’s species richness (*d* = (S–1)/ln(N), where S is total OTU number and N is total reads of each sample) and diversity (Shannon index H′) were calculated. Similarity percentage (SIMPER) analysis of the dissimilarity between *Synechococcus* communities was carried out using Primer 5 (Primer-E Ltd., Plymouth, United Kingdom). The Spearman correlation between *Synechococcus* groups was calculated using R package Corrplot (Wei, 2016). Only the correlations with P-value less than 0.05 were considered as significant and were thus visualized.

### Phylogenetic Analysis of the *rpoC1* and *cpcBA* Sequences

The representative sequences of the 40 most abundant OTUs for the *rpoC1* gene (covered 73.1% of total reads) and *cpcBA* operon (covered 65.7% of total reads) were extracted and aligned with the reference sequences using ClustalW (Thompson et al., 2002) according to their codon structures. Modeltest and maximum likelihood phylogenetic tree construction were done by using Mega 6 (Tamura et al., 2013), in which the model used for the *rpoC1* was GTR+G+I and that for the *cpcBA* operon was TN92+G+I. Bootstrap confidence analysis was carried out with 200 replications for evaluating the robustness of the tree topologies. A heatmap showing the relative abundance of each OTU was generated using iTol (Letunic and Bork, 2007).

### Sequence Submission

All sequences obtained from this study have been deposited in the National Center for Biotechnology Information (NCBI) Sequence Read Archive (SRA) under accession numbers: SRS2048774–SRS2048789 and SRS2048826–SRS2048834 (Supplementary Table S3).

## Results

### Environmental Conditions of the Sampling Stations

As shown in **Figure 1**, strong salinity gradients between the surface and bottom waters were recorded in all sampling stations, except the well-mixed stations A2 and F303. The surface water salinity ranged from 0 to 33 ppt. Along the Pearl River-estuary-coast transect, temperature of the surface waters gradually increased whereas the bottom waters had an opposite pattern. The surface waters had a generally higher temperature and nutrient concentration than the bottom waters. Concentrations of phosphate, NO_3_^-^, NO_2_^-^, and NH_4_^+^ were higher at the stations A2 and A6, which were strongly influenced by the freshwater discharge (**Table 1**). Higher salinity and lower nutrient concentration were recorded in station F303, due to the strong influence of offshore oceanic water.

### *Synechococcus* Abundance

**FIGURE 2:**
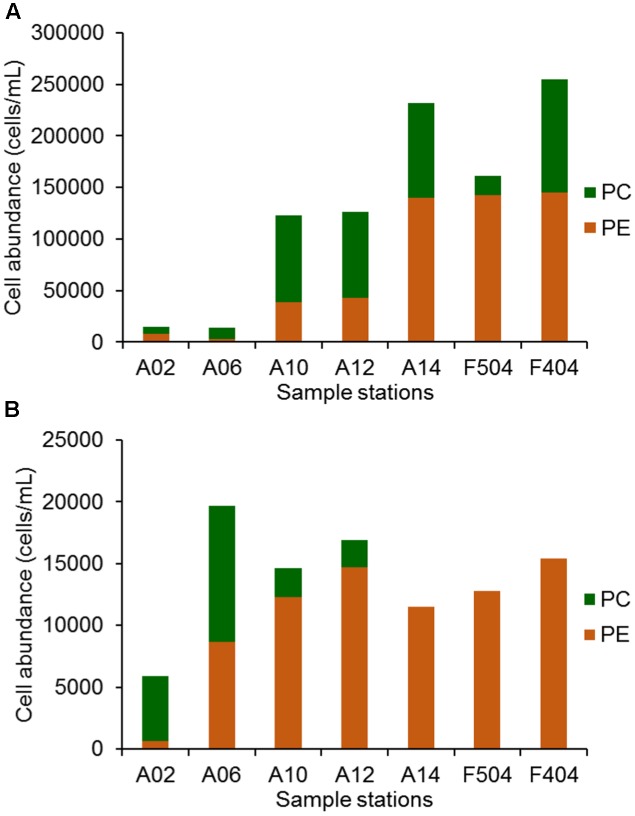
The abundance of PC only and PE containing *Synechococcus* in the surface **(A)** and bottom **(B)** of sampling stations.

*Synechococcus* distributed ubiquitously in the Pearl River estuary and the adjacent coastal waters (**Figure 2**) with abundance ranging from 1.3 × 10^4^ to 2.5 × 10^5^ cells mL^-1^ in the surface waters and from 5.9 × 10^3^ to 2.0 × 10^4^ cells mL^-1^ in the bottom waters. The abundance of *Synechococcus* in the medium and high salinity stations were higher than that of the low salinity stations (A2 and A6). PE-containing *Synechococcus* were found in all samples and its abundance gradually increased with increasing salinity. The highest PE-containing *Synechococcus* abundance was detected in the surface water of A14, F404, and F504 (around 1.4 × 10^5^ cells mL^-1^). PC-only *Synechococcus* were also found in all surface samples, however, they were only detected in the bottom water of stations A02, A06, A10, and A12. In the surface water of stations A10, A12, A14, and F404, PC-only *Synechococcus* abundance could reach 8.0 × 10^4^ cells mL^-1^ (**Figure 2**). In general, PC-only and PE-containing *Synechococcus* were more abundant in the surface waters than the bottom waters at all stations except A6.

### Diversity of *Synechococcus* in the Pearl River Estuary

The number of the *rpoC1* sequences obtained by pyrosequencing was listed in Supplementary Table S3. The diversities of *Synechococcus* assemblages were estimated by the Margalef’s species richness index and Shannon diversity index (Supplementary Figure S1). The surface water of stations A2 and A6, which had low salinities, displayed the lowest *Synechococcus* richness and diversity. The richness and diversity of *Synechococcus* in the bottom waters did not show a large variation, and they were higher in the bottom than the surface waters at all stations, except A10 and A14.

In the phylogenetic tree, all euryhaline (clade VIII, S5.2 and *Cyanobium*) and freshwater *Synechococcus* formed a cluster that is separated from the strictly marine *Synechococcus* clades (Supplementary Figure S2). In the Pearl River estuary, most S5.2 *Synechococcus* were affiliated with WH8007. Freshwater *Synechococcus* were identified into two lineages, FS_I and FS_II, and FS_I had a narrower distribution than FS_II. In the phylogenetic tree, FS_I sequences were affiliated with the uncultured clone sequences from the Tucuru hydroelectric power station reservoir in Brazilian Amazonia, while FS_II sequences were clustered with PS675 and PS676 isolated from Lake Teganuma (Japan) (Supplementary Figure S2). In A2S, almost all of freshwater *Synechococcus* were contributed by FS_II, while that in A6S were mainly from FS_I. OTUs which were belonged to freshwater *Synechococcus*, *Cyanobium* and S5.2 had high relative abundance in the medium salinity waters, while those belonged to clade III, such as OTU1 (contain the most reads), mainly occurred in the medium and high salinity waters. Moreover, S5.3, one of the major group *Synechococcus* in the studied region, had higher relative abundance in the bottom waters. It could be further classified into three subgroups, one was formed by previously reported strains RCC307 and Minos 01, the second by OTU12 and OTU18, and the third by OTU11 and OTU34. However, clade II, which was reported as the dominant *Synechococcus* in tropical/subtropical warm waters by previous studies (Zwirglmaier et al., 2008; Huang et al., 2012; Xia et al., 2017), was not abundant in the studied area. It is surprising that OTU25, which was widely distributed in the bottom water of the Pearl River estuary (A6B, A12B, A14B, F504B, and F404B with relative abundance from 0.01 to 5.5% of sample’s reads), was grouped with clade I *Synechococcus* - a typical cold water lineage.

### Composition of *Synechococcus* Assemblages in the Pearl River Estuary

**FIGURE 3:**
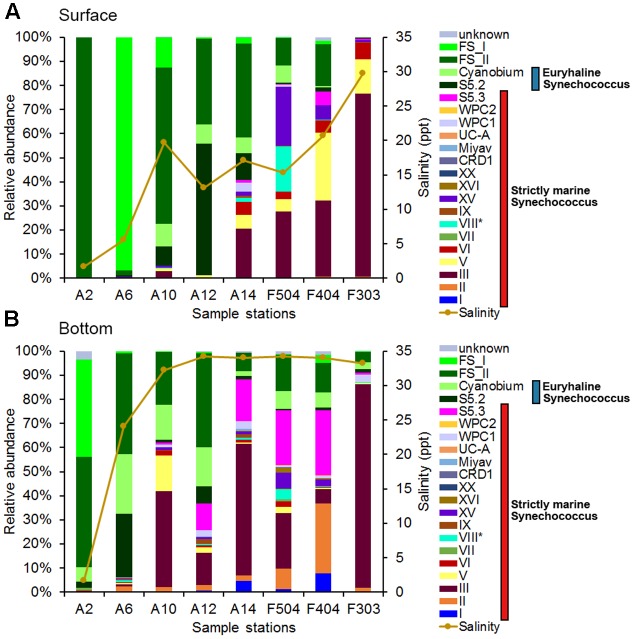
*Synechococcus* community compositions in the surface **(A)** and bottom **(B)** waters based on the *rpoC1* gene. FS_I and FS_II are two phylogenetic groups of fresh *Synechococcus* found in this study (see Supplementary Figure S3). ^∗^S5.1 clade VIII: Euryhaline *Synechococcus*.

Altogether, 21 *Synechococcus* lineages were identified from 16 samples based on *rpoC1* gene (**Figure 3**). Freshwater *Synechococcus* could be detected in all samples, with relative abundance ranging from 0.25 to 99.97% of each samples’ reads (**Figure 3**). More than 98% of the detected cells were freshwater *Synechococcus* in A2S and A6S, where the salinity was lower than 6 ppt. It was found that the dominant *Synechococcus* in the surface waters had shifted with the increase of salinity, from freshwater *Synechococcus* to a combination of freshwater *Synechococcus* and S5.2, and then to S5.1. High relative abundance of clade III was mainly recorded in the A14S, F504S, F404S and F303S, where the salinity is intermediate to high. Clade V, which was also a major S5.1 *Synechococcus* in the studied area, only had high relative abundance in stations F404S and F303S (**Figure 3**).

In general, *Synechococcus* assemblage compositions in the bottom layer were markedly different from that in the surface water layer (**Figure 3**). Freshwater *Synechococcus* largely dominated the bottom water of A2 while *Cyanobium*, S5.2 and 10 clades of S5.1 *Synechococcus* were also detected. Compared with sample A2B, A6B were found with a higher relative abundance of S5.2 and *Cyanobium* instead of the freshwater *Synechococcus*. Moreover, Clade III and S5.3 had a high relative abundance in the bottom water of high salinity stations. Clades I and II were detected in all bottom samples (except A2B and F303B which had no clade I) with relatively low abundance. The highest relative abundance of clade I was detected in F404B, reached 7.89%. Phylogenetic analysis of clade I *rpoC1* sequences showed that OTU71 and OTU85 were affiliated with subclades IC and IA, respectively (Supplementary Figure S3). However, OTU25, which was the most abundant clade I OTU, did not group with reference sequences of reported subclades (Xia et al., 2017) (82%–86% nt identity to the subclades’ representative sequences and 99% to uncultured *Synechococcus* RFLP-type S14 (AJ584725.1)) and may belong to a novel subclade (Supplementary Figure S3).

**FIGURE 4:**
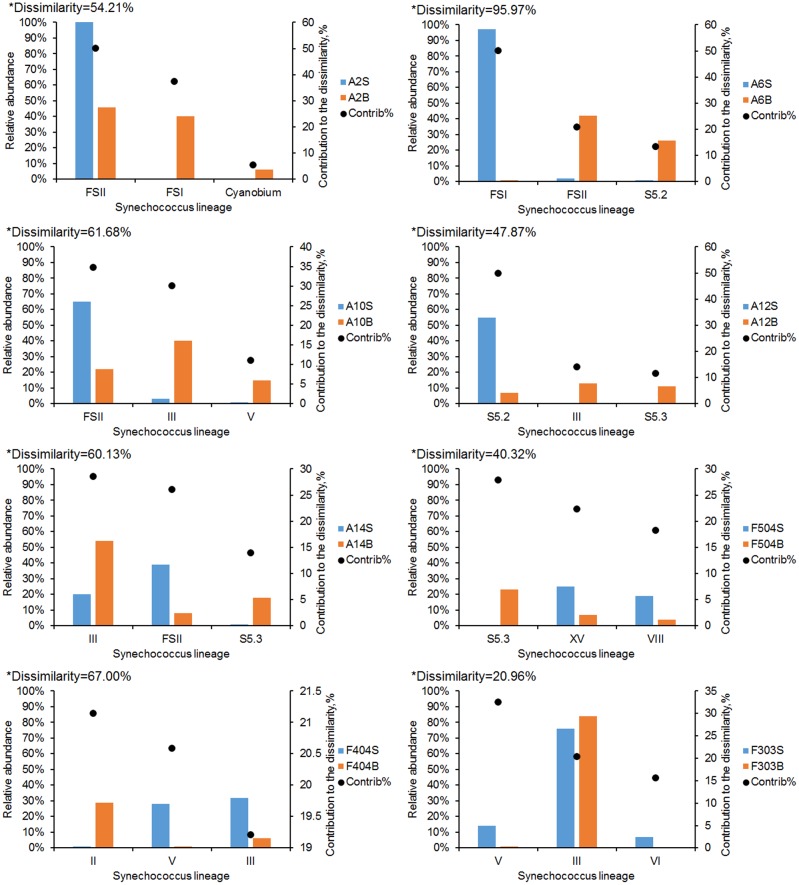
SIMPER analysis of the dissimilarity between *Synechococcus* communities in the surface and bottom waters in each station. Bar charts show the relative abundance of the three *Synechococcus* lineages that contributed most to the dissimilarity of communities. ^∗^The dissimilarity between the surface and bottom *Synechococcus* communities.

The dissimilarity between surface and bottom *Synechococcus* communities was analyzed using SIMPER analysis (**Figure 4**). The lowest dissimilarity (20.96%) was detected at station F303, where water was well mixed. The dissimilarity in the stratified stations ranged from 40.32 to 95.97%. The highest dissimilarity occurred at station A6, which was mainly contributed by FS_I, FS_II and S5.2. FS_II and clade III were the major contributors of the dissimilarity at stations A10 and A14, where FS_II had higher relative abundance in the surface waters, while clade III were relatively more abundant in the bottom. S5.3, which was mainly distributed in the bottom waters, was also a major contributor to the dissimilarity at stations A12, A14, and F504.

**FIGURE 5:**
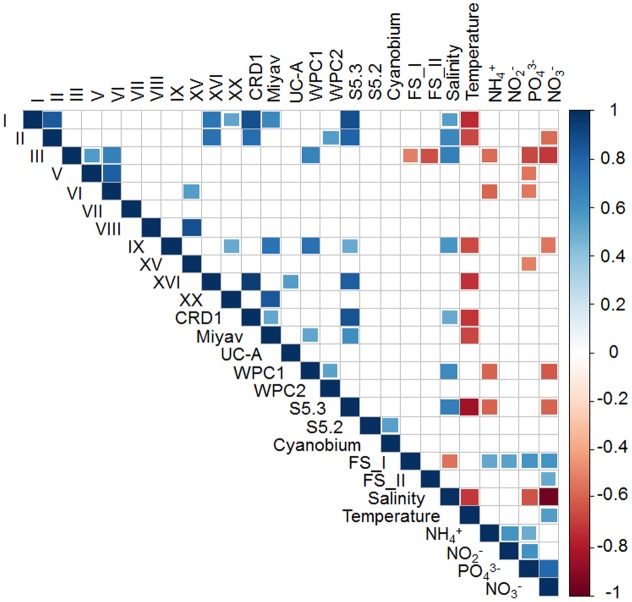
Spearman rank correlation between *Synechococcus* clades and environmental factors. Only significant correlations (*P* < 0.05) are shown.

Spearman’s correlation coefficients were calculated between the *Synechococcus* lineages and environmental factors (**Figure 5**). Being significantly correlated to each other positively, clades I, II, XVI, CRD1, and S5.3 were inversely correlated with temperature and were mainly distributed in the bottom layer. Besides, freshwater *Synechococcus* FS_I was strongly negatively associated with salinity and positively related with nutrient concentrations, which was contrasting to clades III, IX, WPC1, and S5.3 which preferred high salinity and low NO_3_^-^ environment. It was noted that the *Synechococcus* lineages with the highest relative abundance in the Pearl River estuary, clade III and freshwater *Synechococcus* (FS_I and FS_II), were negatively correlated to each other, which indicates an opposite distribution pattern. On the other hand, euryhaline *Synechococcus* S5.2 was highly positively correlated with *Cyanobium*, which suggests that they shared similar niches.

### *Synechococcus* Assemblage Harboring in the Surface and Bottom Waters had Different Pigment Compositions

Based on the successful amplification and sequencing of the *cpcBA* operon sequences from eight samples (the other samples did not amplify) (Supplementary Table S3), 4 well-separated clusters were formed in the phylogenetic tree (Supplementary Figure S4). Although type 1, 2, and 3b *Synechococcus* could be easily classified by the sequencing of *cpcBA* sequence, PUB containing *Synechococcus* type 3a, 3c, 3d, and 3f (recently defined by Mahmoud et al., 2017) could not be distinguished from each other (Supplementary Figure S4). Type 3 sequences from S5.3 formed a clade (hereafter named S5.3-Type 3) and were separated from the clade formed by those from S5.1 (hereafter named S5.1-Type 3). The phylogenetic tree also shows that most of the type 1 OTUs were affiliated with PS673 and PS676. Only 1 of the 40 most abundant OTUs was identified as S5.3-Type 3, which was mainly distributed in the bottom waters.

**FIGURE 6:**
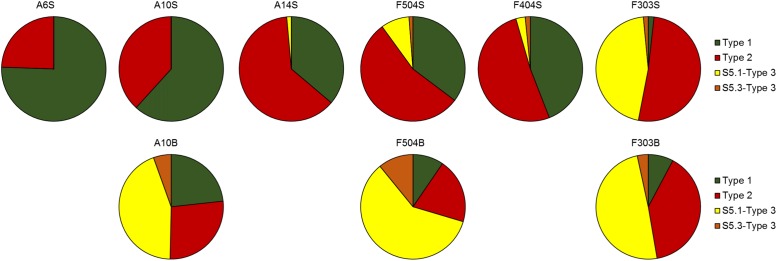
*Synechococcus* pigment compositions in the surface and bottom samples revealed by sequencing the *cpcBA* operon sequences.

Distributed widely in the surface samples (**Figure 6**), proportion of type 1 decreased gradually while type 2 increased with increasing salinity. Only a small portion of *Synechococcus* detected was identified as type 3 at the stations of lowest salinity (A6S and A10S), comparing to more than 44.8% in the oceanic water (F303S). Besides, *Synechococcus* pigment compositions in the surface and bottom waters at the two stratified stations (A10 and F504) were remarkably different. While station A10B was dominated by S5.1-type 3 *Synechococcus*, A10S were mainly dominated by type 1 and type 2. Moreover, the relative abundance of type 3 *Synechococcus* was also greatly higher in the bottom than in the surface at station F504. S5.3-Type 3, which was not abundant in the surface waters, had higher relative abundance in the bottom water of stations A10 and F504. However, in well mixed station F303, similar *Synechococcus* pigment composition in the surface and bottom layers were detected, which were composed of more type 2 and 3 cells than type 1.

## Discussion

The abundance and diversity of *Synechococcus* were extensively studied in various marine environments, from oligotrophic open ocean to subtropical coastal and estuarine waters. However, none of the studies systematically reported the *Synechococcus* diversities in the salt wedge estuaries. Here, we used flow cytometric analysis and pyrosequencing method to assess the abundance, pigment diversity (based on the *cpcBA* operon) and taxonomic diversity (based on the *rpoC1* gene) of *Synechococcus* in the Pearl River estuary, a typical salt wedge estuary in summer. Our results revealed that *Synechococcus* were highly abundant in this subtropical estuary, with a clear spatial variation in phylogenetic composition and pigment diversity along the surface salinity gradient, as well as between the surface and bottom waters.

Previous study has suggested that next generation sequencing methods with high sensitivity could yield more insights into the *Synechococcus* community composition than the traditional clone library method and flow cytometry approach (Xia et al., 2015). Consistently, in the present study, PC only *Synechococcus* were detected in all samples by using the pyrosequencing method while they could not be detected in some bottom samples by applying the flow cytometry approach. Moreover, using sequencing method, different pigment types and phylogenetic groups can be identified, providing more information about the composition of *Synechococcus* community.

### Abundance of *Synechococcus* along the Salinity Gradient of River Plume

High abundance of *Synechococcus* (up to 2.5 × 10^5^ cells mL^-1^ in surface waters) was observed in the Pearl River estuary in July, which was higher than most other marine environments (Flombaum et al., 2013), suggesting that *Synechococcus* were important primary producers in the subtropical river-impacted coastal water (Qiu et al., 2010). Spatial variations in *Synechococcus* abundance and the distribution of *Synechococcus* groups observed in the Pearl River estuary (**Figure 2**) was consistent with the studies carried out in other estuaries, such as Chesapeake Bay (Wang et al., 2011) and Zuari estuary (Rajaneesh and Mitbavkar, 2013), which have also displayed increasing *Synechococcus* abundance along the salinity gradient. Low salinity (Wang et al., 2011; Rajaneesh and Mitbavkar, 2013; Xia et al., 2015) and light limitation (Harrison et al., 2008) could be the reasons of low *Synechococcus* abundance in the freshwater-dominated estuarine water.

### Shifts in Phylogenetic Composition and Pigment Diversity of *Synechococcus* Assemblages Along the Salinity Gradient in Subtropical River-Estuary-Shelf

The phylogenetic compositions of *Synechococcus* assemblage (assessed using the *rpoC1* gene) varied along the salinity gradient. It is not surprising that freshwater *Synechococcus* were dominant in the inner field of the estuary (A2S and A6S), where turbid river water reigns. However, it was shown in the phylogenetic analysis that most of the *Synechococcus* detected in these two samples belonged to two distinct OTUs, OTU2 and OTU3, which suggests the niche differentiation among subgroups of freshwater *Synechococcus*. Freshwater *Synechococcus* were also abundant in A10S, A12S, and A14S, of which the salinity ranged from 13.1 to 19.7 ppt. This observation contrasted with the study in the Chesapeake Bay, the largest estuary in the United States, where freshwater *Synechococcus* are rare (Chen et al., 2006). Apart from the freshwater *Synechococcus*, euryhaline *Synechococcus* S5.2, and *Cyanobium* were also abundant in the intermediate salinity water. Their preferences of higher salinity environments compared with the freshwater *Synechococcus* agreed with the finding of a previous study that S5.2 *Synechococcus* has a high ability to deal with low salinity stress but requires elevated salinity for growth (Wang et al., 2011). Co-occurrence of S5.2 and *Cyanobium* was also reported by a study conducted in Hong Kong water (Xia et al., 2015) and Baltic Sea blackish water (Celepli et al., 2017), suggesting the two *Synechococcus* lineages have similar physiological and ecological characteristics. However, the Spearman analysis did not show any strong correlation between the distribution of these two lineages and any measured environmental factors (**Figure 5**).

The proportion of S5.1 lineages increased with salinity (**Figure 3**). Celepli et al. (2017) reported that in the southern Baltic Sea, *Synechococcu*s community transitioned from being dominated by euryhaline *Synechococcus* and *Cyanobium* to a mix of euryhaline and marine *Synechococcus* strains of S5.1 taking place at a salinity of 13–16 ppt. Similarly, our study showed that the transition occurred at salinity around 15 ppt in the Pearl River estuary (**Figure 3**). In the Baltic Sea coastal water, *Synechococcus* community is dominated by cold water clades I and IV, while the brackish and saline waters in the Pearl River estuary was widely dominated by the clade III. High relative abundance of clade III found in both the brackish and saline waters is consistent with the observation in the ECS (Choi et al., 2011; Xia et al., 2017). However, this is in contrast with the report in the Mediterranean Sea where clade III *Synechococcus* was mainly found in high salinity, oligotrophic, and phosphate-depleted water (Mella-Flores et al., 2011). The contrasting results observed by different studies were accounted by the fact that clade III contains several ecologically significant taxonomic units (ESTUs) with distinct niche preferences (Farrant et al., 2016). Furthermore, a strongly positive correlation of clade III and WPC1 (first found in the East China Sea and the Japan Sea (Choi and Noh, 2009)) was shown in the correlation analysis (**Figure 5**), which coincides with the finding of co-occurrence of clade III and WPC1 reported by previous studies (Choi et al., 2015; Xia et al., 2017). Besides that, clades V and VI, which overall distribution is not well understood, also co-occurred with clade III. Clades III, V and, VI and III were negatively related to nutrient concentrations, suggesting they have preferences of oceanic environment. Clade XV, which mainly occur between 30° and 35°N/S (Huang et al., 2012; Sudek et al., 2015) and in upwelling regions (Sohm et al., 2016), was also distributed in the surface of F504 with relatively high relative abundance. Although previous studies reported that clade II is the dominant clade in the tropical/subtropical warm water (Zwirglmaier et al., 2008), we found this clade not abundant in the Pearl River estuary and its adjacent coastal water. Low abundance of clade II in this area may be due to the fact that clade II has fewer regulators (Palenik et al., 2006) to adapt to such dynamic and highly variable estuary-shelf environment. As a single *Synechococcus* clade can possess different pigment types, it is impossible to identify pigment types based on housekeeping genes, such as *16S rRNA* and *rpoC1* (Haverkamp et al., 2009; Everroad and Wood, 2012; Xia et al., 2017). Instead, the analysis of *cpcBA* operon (encoding phycocyanin) and *cpeBA* operon (encoding phycoerythrin) were applied to study *Synechococcus* pigment diversity in marine environments. Using the *cpeBA* sequence, a recent study found four groups of *Synechococcus* pigment types: 2, 3a, 3dA and the combination of 3c and 3dB can be identified (Xia et al., 2017). However, this gene marker cannot be applied to identify PC-only *Synechococcus* because they do not have the *cpeBA* operon. Hence, in this study, we used the *cpcBA* operon for studying pigment diversity in the Pearl River estuary. Haverkamp et al. (2009) suggested that the high phylogenetic resolution provided by the *cpcBA* operon is useful to assess the microdiversity of *Synechococcus* strains. Phylogenetically, this gene marker is capable of differentiating type 1, 2 and type 3 *Synechococcus*, while subtypes of type 3 (3a, 3c, and 3d) cannot be distinguished (Supplementary Figure S4). Yet, we found that this gene marker allows us to assign type 3 to S5.1 or S5.3 (Supplementary Figure S4). Studies have reported that different *Synechococcus* pigment types often co-occur in a marine environment, while one phenotype generally predominates (Haverkamp et al., 2009). Consistently, we found co-occurrence of *Synechococcus* pigment types in our samples. Dominant pigment type shifted from type 1 to type 3 along the high turbid freshwater-dominated estuary to the shelf water, on top of the relatively abundant of the widely occurring type 2 *Synechococcus* across the whole study area. Such a distribution pattern supports the point that underwater light spectral properties have a strong selective pressure on *Synechococcus* populations (Vörös et al., 1998; Six et al., 2007; Stomp et al., 2007; Xia et al., 2017).

### Markedly Different *Synechococcus* Assemblages Harboring in the Surface and Bottom Waters of the Salt Wedge Estuary

The partition of *Synechococcus* lineages along depth is not as strong as the horizontal scale in marine water (Zwirglmaier et al., 2008). Therefore, *Synechococcus* assemblage composition in the surface water is generally representing the community at lower depth (Sohm et al., 2016). Indeed, *Synechococcus* assemblage had similar compositions in the surface and deep layers of the oceanic station F303, where strong mixing occurred. However, the assemblage displayed vertical differentiation in the stratified water. The surface water, which was a mixture of freshwater and marine water, was characterized with low salinity and high nutrient (Harrison et al., 2008). This environment would favor the selection of euryhaline strains which have a higher requirement of nutrients. On the other hand, the deep layer features high salinity but relatively low nutrient marine water (Harrison et al., 2008) which is suitable for the growth of strictly marine *Synechococcus*. For example, in the surface water of A10 and A12 euryhaline S5.2 *Synechococcus* had high relative abundance, while S5.1 *Synechococcus* had high proportion in the bottom waters.

Interestingly, S5.3, a minor group in marine environments, was widely detected from the bottom layer of stratified stations. S5.3 has at least six clades and shows depth partitioning (Huang et al., 2012). S5.3-I, represented by RCC307, is mainly present in surface water layer, while S5.3-II, -IV, -V, and –VI prevail in the medium to low light layer (Huang et al., 2012). Based on the *rpoC1* gene sequence, we found that S5.3 in the Pearl River estuary was not as diverse as in the open ocean and was abundant in the bottom layer (Supplementary Figure S2). Their distribution was significantly positively related to salinity while negatively correlated with temperature, NH_4_^+^ and NO_3_^-^ (**Figure 5**). This is in agreement with Hashimoto et al.’s (2012) observation that S5.3 mainly occurs in deep waters. Apart from S5.3, we observed that clade I also widely occurred in the bottom layer where temperature could exceed 23°C. This is in contrast with the conclusion of previous studies that clade I is restricted in high latitude cold water (Zwirglmaier et al., 2008; Huang et al., 2012; Sohm et al., 2016). A recent study reported that clade I contains at least six subclades with different thermal preferences (Xia et al., 2017). Consistently, only warm water subclades, IA and IC (see Figure 8 in Xia et al., 2017), were detected in the Pearl River estuary (Supplementary Figure S3). Besides these two subclades, OTU25, the most abundant clade I OTU, did not cluster with all reported subclades (Xia et al., 2017), but formed another novel subclade (subclade IG) (Supplementary Figure S3). The fact that subclade IG, defined by this study, was mainly distributed in deep water may be the reason why this subclade has not previously been detected. Huang et al. also detected clade I in the South China Sea at relatively deep layers of 75 and 100 m depth with relatively high abundance by sequencing 16S-23S rRNA internal transcribed spacer (ITS) (Huang et al., 2012). This suggests that clade I may be globally distributed and some subclades are specifically distributed in the deep water of tropical/subtropical region.

## Conclusion

The river-estuary-shelf continuum is a highly complex system, which provides a wide array of niches for a highly diverse *Synechococcus* assemblage ranging from freshwater *Synechococcus* to euryhaline and strictly marine *Synechococcus*. Our data suggest that *Synechococcus* lineages have markedly different abilities to deal with environmental variations. In the estuary, salinity is an important factor influencing the distribution of *Synechococcus* groups. More studies are needed to reveal the mechanisms involved in salinity tolerance. The fact that high abundance of clade III occurs in the brackish coastal water may revise our previous understanding that clade III prefers oligotrophic oceanic water. Our results further reveal that clade I and S5.3 contain subgroups that have different niches. Further studies should focus on isolation of *Synechococcus* strains from the studied area and the physiological traits of clades I, III, and S5.3 strains. Moreover, to uncover more details about the distribution of *Synechococcus* in the salt wedge estuary, high resolution sampling (both vertical and horizontal) need to be conducted in future studies.

## Author Contributions

HL designed the experiment. XX and WG performed the experiments. Data were analyzed by XX in collaboration with WG and HL. XX and HL wrote the manuscript. ST attended the cruise and collected FM and DNA samples. All authors reviewed and approved the final version of the manuscript.

## Conflict of Interest Statement

The authors declare that the research was conducted in the absence of any commercial or financial relationships that could be construed as a potential conflict of interest.

## Footnotes

1 http://roscoff-culture-collection.org/strains/shortlists/taxonomic-groups/marine-synechococcus

2 http://www.mothur.org/wiki/Download_mothur

3 https://www.ncbi.nlm.nih.gov/

4 http://roscoff-culture-collection.org/strains/shortlists/taxonomic-groups/marine-synechococcus
