# Supplementary material for: Synechococcus Assemblages across the Salinity Gradient in a Salt Wedge Estuary
Source: Front Microbiol. 2017 Jul 6;8:1254. doi: 10.3389/fmicb.2017.01254 (PMC5498518; doi:10.3389/fmicb.2017.01254)
Supplement: Supplementary file 1 [file Data_Sheet_1.docx]

Supplementary Material

# *Synechococcus* assemblages across the salinity gradient in a salt wedge estuary

**Xiaomin Xia, Wang Guo, Shangjin Tan, Hongbin Liu***

*** Correspondence:** Corresponding Author: liuhb@ust.hk

# Supplementary Figures and Tables

## Supplementary Figures


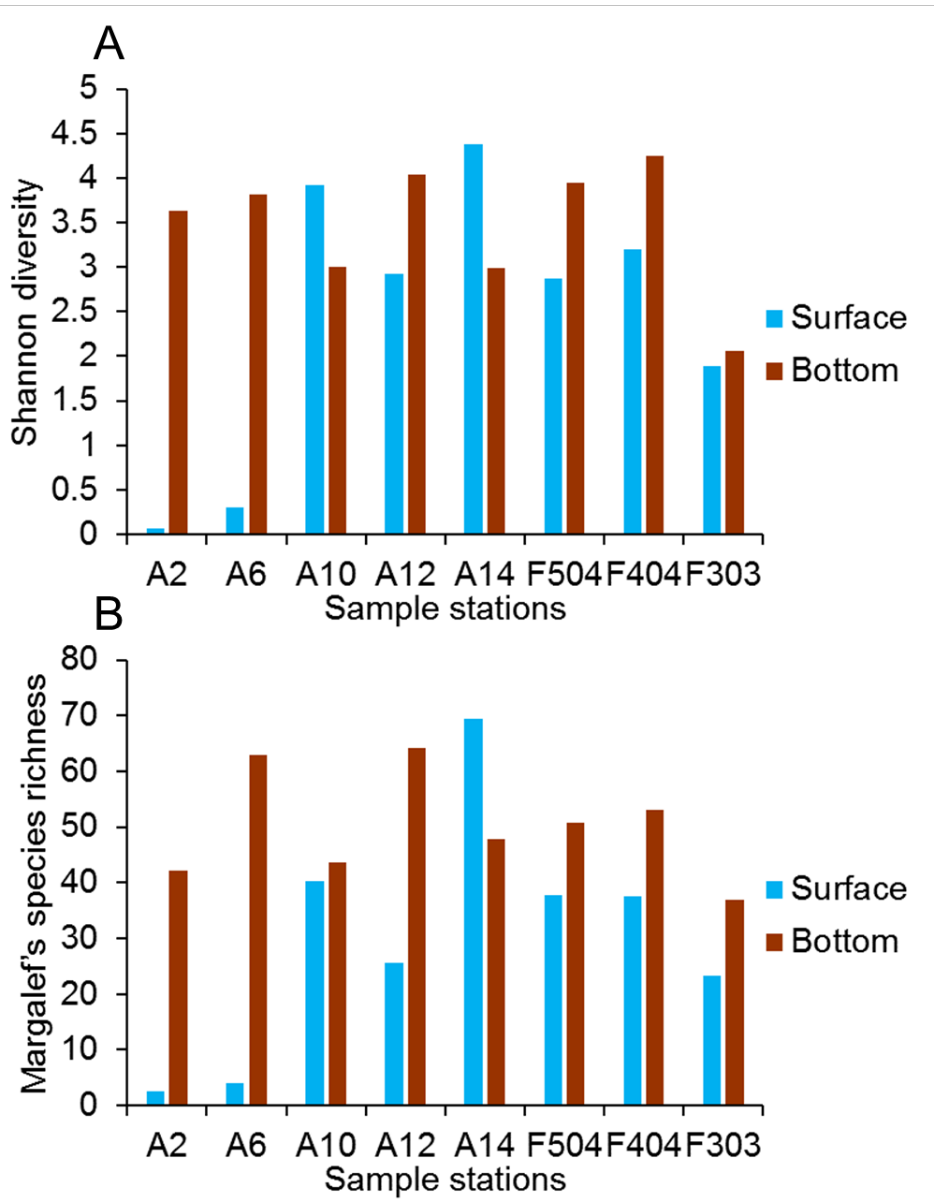


Fig. S1 The Shannon diversity (A) and Margalef’s species richness (B) of *Synechococcus* communities at each station along the salinity gradient.


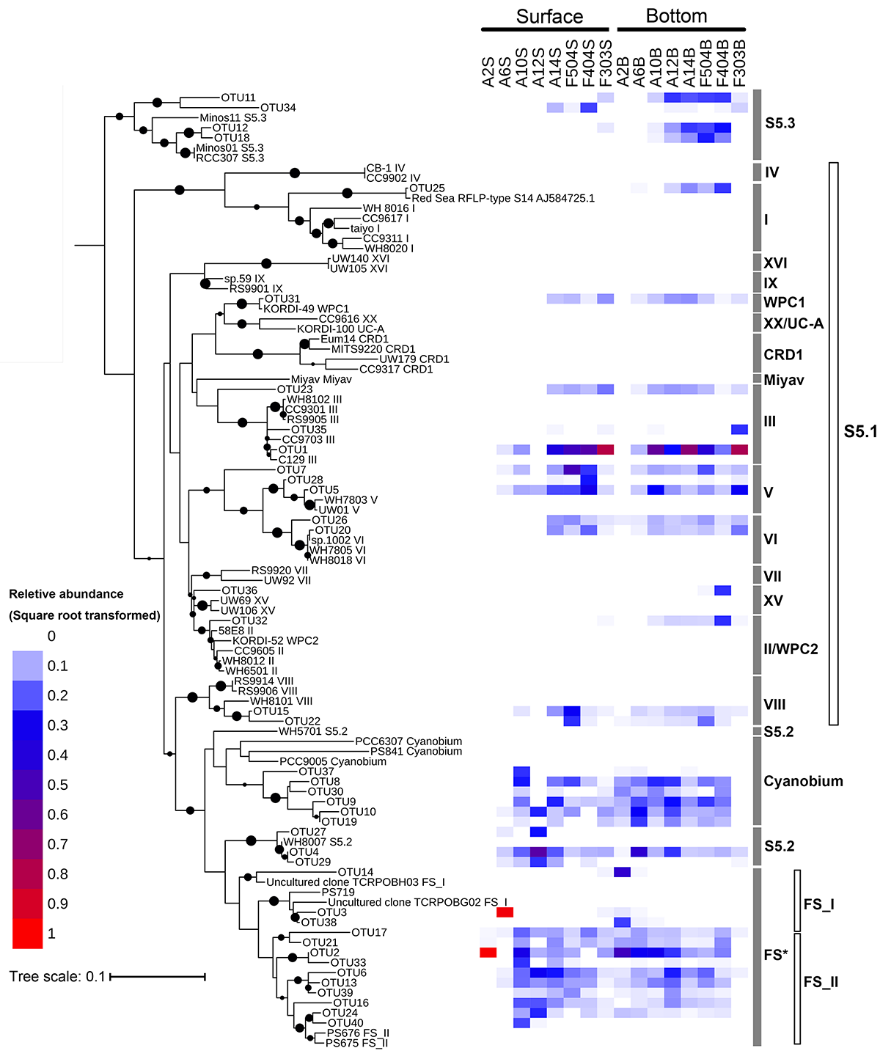


Fig. S2 Maximum likelihood phylogenetic tree of the 40 most abundant *rpoC1* OTUs across all samples. Heatmap on right-hand side shows the relative abundance of OTUs in each library (square root transformed). Only nodes with bootstrap values higher than 50% are shown. *Freshwater *Synechococcus*. The NCBI accession number of Uncultured clone TCRPOBG01, Uncultured clone TCRPOBG02 and Red Sea RFLP-type S14 is FJ849958.1, FJ850032.1 and AJ584725.1, respectively.


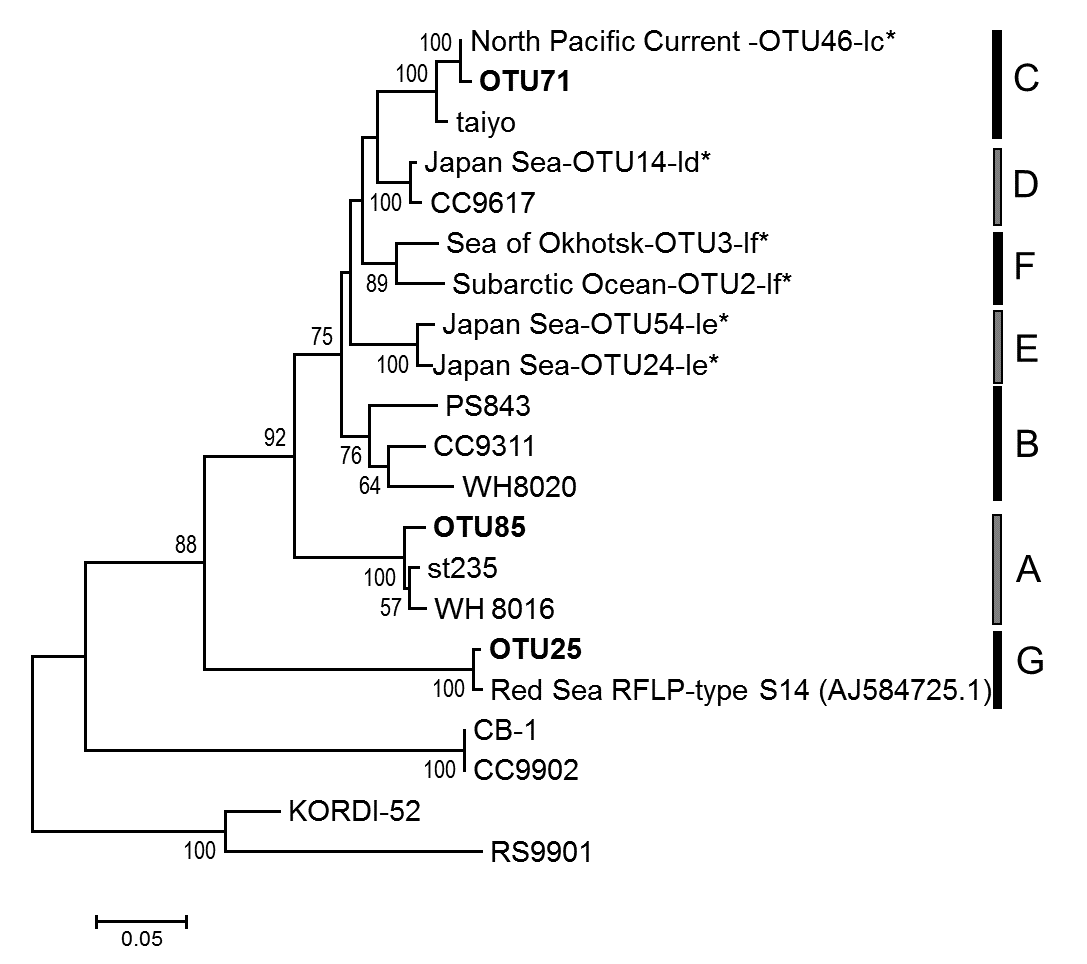


Fig. S3 Maximum-likelihood phylogenetic tree of clade I *Synechococcus*. Only clade I OTUs in the 100 most abundant *rpoC1* OTUs across all samples (in bold) were used to construct the tree. Only nodes with bootstrap values higher than 50% are shown. Letters A–F on right-hand side correspond to different subclades within clade I which were defined by Xia et al. (2017). *reference sequences from Xia et al. (2017).


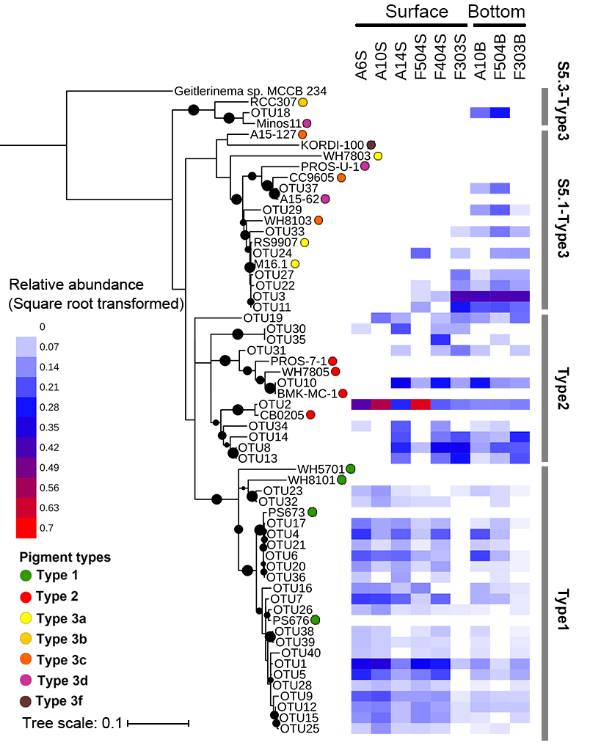


Fig. S4 Phylogenetic analysis of representative *cpcBA* operon sequences of the 40 most abundant OTUs. Heatmap indicates the relative abundance of each OTU (square root transformed). Right bars indicate the clusters formed by different pigment types. Bootstrap values larger than 50% were showed. As there were three copies of the *cpcBA* operon in the genomic sequence of type 1 *Synechococcus* (Six et al., 2007), the number of resulting type 1 sequences was divided by three in calculating the relative abundance of each *Synechococcus* pigment type. Type 3f was recently defined by Mahmoud et al. (Mahmoud et al., 2017).

## Supplementary Tables

Table S1 Reference *rpoC1* sequences used for local blast.

| Representative strain | clade | cluster | Accession number |
| --- | --- | --- | --- |
| *Synechococcus* sp. WH8016 | I | Subcluster 5.1 | GU990533.1 |
| *Synechococcus* sp. CC9617 | I | Subcluster 5.1 | AF154562.1 |
| *Synechococcus* sp. WH8020 | I | Subcluster 5.1 | AF323594.1 |
| Red Sea-RFLP type S14 | I | Subcluster 5.1 | AJ584725.1 |
| *Synechococcus* sp. WH8012 | II | Subcluster 5.1 | JQ421062.1 |
| *Synechococcus* sp. miyar | II | Subcluster 5.1 | AF448102.1 |
| *Synechococcus* sp. RS9907 | II | Subcluster 5.1 | AJ621007.1 |
| *Synechococcus* sp. RS9902 | II | Subcluster 5.1 | AJ621004.1 |
| *Synechococcus* sp. CC9605 | II | Subcluster 5.1 | AF154560.1 |
| *Synechococcus* sp. WH8102 | III | Subcluster 5.1 | AF153336.1 |
| *Synechococcus* sp. CC9703 | III | Subcluster 5.1 | AF153338.1 |
| *Synechococcus* sp. CC9702 | III | Subcluster 5.1 | AF153337.1 |
| *Synechococcus* sp. CC9301 | III | Subcluster 5.1 | AF153332.1 |
| *Synechococcus* sp. WH8103 | III | Subcluster 5.1 | L34063.1 |
| *Synechococcus* sp. CB1 | IV | Subcluster 5.1 | GU990581.1 |
| *Synechococcus* sp. CC9902 | IV | Subcluster 5.1 | ABB25574. |
| *Synechococcus* sp. UW01 | V | Subcluster 5.1 | AJ621020.1 |
| *Synechococcus* sp. WH7803 | V | Subcluster 5.1 | L34061.1 |
| *Synechococcus* sp. WH8018 | VI | Subcluster 5.1 | AJ621015.1 |
| *Synechococcus* sp.1002 | VI | Subcluster 5.1 | AF448082.1 |
| *Synechococcus* sp. WH7805 | VI | Subcluster 5.1 | L34062.1 |
| *Synechococcus* sp. RS9920 | VII | Subcluster 5.1 | AJ621012.1 |
| *Synechococcus* sp. UW92 | VII | Subcluster 5.1 | JQ421053.1 |
| *Synechococcus* sp. WH8101 | VIII | Subcluster 5.1 | JQ421065.1 |
| *Synechococcus* sp. RS9906 | VIII | Subcluster 5.1 | JQ421058.1 |
| *Synechococcus* sp. RS9901 | IX | Subcluster 5.1 | AJ621003.1 |
| *Synechococcus* sp. 59 | IX | Subcluster 5.1 | AF448100.1 |
| *Synechococcus* sp. UW179 | CRD1 | Subcluster 5.1 | JQ421060.1 |
| *Synechococcus* sp. CC9317 | CRD1 | Subcluster 5.1 | AF013609.1 |
| *Synechococcus* sp. MITS9220 | CRD1 | Subcluster 5.1 | JQ421059.1 |
| *Synechococcus* sp. 9616 | XX | Subcluster 5.1 | AF154561.1 |
| *Synechococcus* sp. UW106 | XV | Subcluster 5.1 | JQ421054.1 |
| *Synechococcus* sp. UW105 | XVI | Subcluster 5.1 | JQ421055.1 |
| *Synechococcus* sp. UW140 | XVI | Subcluster 5.1 | JQ421061.1 |
| *Synechococcus* sp. miyav | Undescribed clade miyav | Subcluster 5.1 | AF448103.1 |
| *Synechococcus* sp. KORDI-100 | UC-A | Subcluster 5.1 | CP006269.1 |
| *Synechococcus* sp. KORDI-52 | WPC2 | Subcluster 5.1 | CP006271.1 |
| *Synechococcus* sp. KORDI-49 | WPC1 | Subcluster 5.1 | CP006270.1 |
| *Synechococcus* sp. WH5701 | S5.2 | Subcluster 5.2 | AF448108.1 |
| *Synechococcus* sp. WH8007 | S5.2 | Subcluster 5.2 | AF448107.1 |
| *Synechococcus* sp. Minos11 | Minos11 | Subcluster 5.3 | AJ621021.1 |
| *Synechococcus* sp. RCC307 | RCC307 | Subcluster 5.3 | CT978603.1 |
| *Synechococcus* sp. Minos01 | Minos01 | Subcluster 5.3 | AJ621022.1 |
| *Synechococcus* sp. PS676 | FS | Freshwater PC-type | AF245135.1 |
| *Synechococcus* sp. PS719 | FS | Freshwater PE-type | AF245141.1 |
| *Synechococcus* sp. PCC9005 | *Cyanobium* | *Cyanobium* | AF245160.1 |
| *Synechococcus* sp. PCC6307 | *Cyanobium* | *Cyanobium* | U52342.1 |
| *Synechococcus* sp. PS841 | *Cyanobium* | *Cyanobium* | AF448092.1 |
| *Synechocystis* sp. PCC7002 |  | *Synechocystis* | U52345.1 |
| *Prochlorococcus* str. MIT9301 |  | *Prochlorococcus* | NC009091.1 |
| *Prochlorococcus* str. MIT9515 |  | *Prochlorococcus* | NC008817.1 |
| *Prochlorococcus* str. NATL1A |  | *Prochlorococcus* | NC008819.1 |
| *Prochlorococcus* str. MIT9211 |  | *Prochlorococcus* | NC009976.1 |
| *Prochlorococcus* str. MIT9303 |  | *Prochlorococcus* | NC008820.1 |

Table S2 Reference *cpcBA* operon sequences used for local blast.

| Representative strain | Pigment type | Accession number |
| --- | --- | --- |
| *Synechococcus* sp. WH5701 | 1 | NZ_CH724159.1 |
| *Synechococcus* sp. ACT0616 | 1 | HQ859452.1 |
| *Synechococcus* sp. PS673 | 1 | AF223434.1 |
| *Synechococcus* sp. PS676 | 1 | AF223439.1 |
| *Synechococcus* sp. WH8101 | 1 | KF528825.1 |
| *Synechococcus* sp. RS9917 | 1 | NZ_CH724158.1 |
| *Synechococcus* sp. A15-44 | 2 | KF528820.1 |
| *Synechococcus* sp. PROS-7-1 | 2 | KF528821.1 |
| *Synechococcus* sp. BMK-MC-1 | 2 | KF528822.1 |
| *Synechococcus* sp. CB0205 | 2 | NZ_ADXM01000022.1 |
| *Synechococcus* sp. WH7805 | 2 | NZ_CH724168.1 |
| *Synechococcus* sp. RS9907 | 3a | KF528813.1 |
| *Synechococcus* sp. RS9901(RCC540) | 3a | FR715491.1 |
| *Synechococcus* sp. M16.1 | 3a | KF528812.1 |
| *Synechococcus* sp. WH7803 | 3a | X59809 |
| *Synechococcus* sp. RCC307 | 3b | CT978603.1 |
| *Synechococcus* sp. WH8103 | 3c | KF528809.1 |
| *Synechococcus* sp. A15-127 | 3c | KF528804.1 |
| *Synechococcus* sp. CC9605 | 3c | CP000110.1 |
| *Synechococcus* sp. Minos11 | 3d | KF528826.1 |
| *Synechococcus* sp. CC9902 | 3d | CP000097.1 |
| *Synechococcus* sp. PROS-U-1 | 3d | KF528817.1 |
| *Synechococcus* sp. A15-62 | 3d | KF528815.1 |
| *Synechococcus* sp. KORDI-100 | 3f | CP006269.1 |

Table S3 No. of raw and qualified *rpoC1* gene sequence and *cpcBA* operon sequence

| Sample | No. of raw sequences (rpoC1) | No. of qualified sequences (rpoC1) | accession number (rpoC1) | No. of raw sequences (cpcBA) | No. of qualified sequences (cpcBA) | acession number (cpcBA) |
| --- | --- | --- | --- | --- | --- | --- |
| A2B | 9680 | 5512 | SRS2048787 |  |  |  |
| A2S | 10133 | 9197 | SRS2048789 |  |  |  |
| A6B | 14665 | 8937 | SRS2048786 |  |  |  |
| A6S | 9489 | 2374 | SRS2048788 | 2893 | 1109 | SRS2048834 |
| A10B | 13628 | 7592 | SRS2048784 | 2057 | 850 | SRS2048832 |
| A10S | 9790 | 5606 | SRS2048785 | 914 | 397 | SRS2048833 |
| A12B | 14953 | 8301 | SRS2048782 |  |  |  |
| A12S | 8820 | 5296 | SRS2048783 |  |  |  |
| A14B | 13121 | 8846 | SRS2048780 |  |  |  |
| A14S | 10046 | 4621 | SRS2048781 | 1056 | 362 | SRS2048831 |
| F303B | 10893 | 7252 | SRS2048774 | 3061 | 1020 | SRS2048826 |
| F303S | 7413 | 5127 | SRS2048775 | 1448 | 408 | SRS2048827 |
| F404B | 11578 | 6630 | SRS2048776 |  |  |  |
| F404S | 10707 | 6679 | SRS2048777 | 1610 | 589 | SRS2048828 |
| F504B | 10217 | 5509 | SRS2048778 | 824 | 350 | SRS2048829 |
| F504S | 11938 | 7874 | SRS2048779 | 2163 | 835 | SRS2048830 |
